# Preference for low-coordination sites by adsorbed CO on small platinum nanoparticles

**DOI:** 10.1039/c9na00499h

**Published:** 2020-01-23

**Authors:** M. Jakir Hossain, M. M. Rahman, Md. Jafar Sharif

**Affiliations:** Forest Chemistry Division, Bangladesh Forest Research Institute Chittagong-4211 Bangladesh smjakir080@gmail.com; Department of Chemistry, University of Dhaka Dhaka-1000 Bangladesh; Department of Chemistry, Military Institute of Science & Technology (MIST) Mirpur Dhaka-1216 Bangladesh

## Abstract

FTIR spectra of ^12^CO adsorbed on poly(vinylpyrrolidone) (PVP)-stabilized colloidal platinum at room temperature were acquired and studied. Two new bands, at 2021 cm^−1^ and 1994 cm^−1^, were observed for the first time and were assigned to the stretching vibrations of CO linearly adsorbed on the Pt surface at edge and corner sites, respectively. The relative intensities of these two bands were found to vary with the coverage of CO, where the smallest particles showed the highest intensity, corresponding to the relative quantities of edge and corner sites per unit surface. The vibrational spectra signals reported for terrace sites on colloidal Pt red-shifted as the particle size was decreased, which showed the electronic interactions between the Pt surface and PVP, with PVP acting as an electron donor.

## Abstract 2

Successfully synthesized PVP stabilized Pt NPs from 1.4 to 4.8 nm. Two new bands at 2021 cm^−1^ and 1994 cm^−1^ were observed in the FTIR spectra of adsorbed ^12^CO on Pt NPs which are assigned to the edge and corner sites at Pt surface respectively.

## Graphical abstract

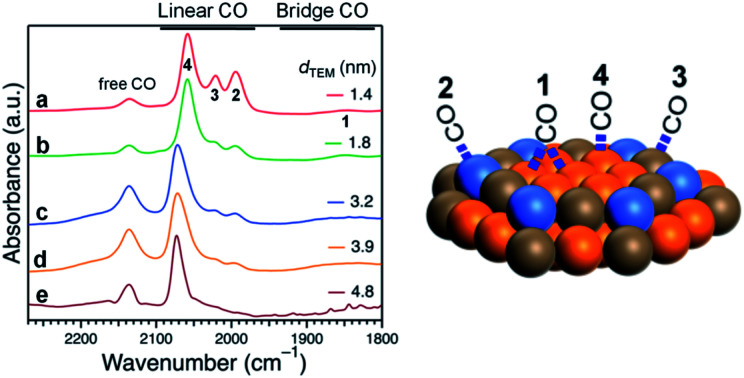

## 1. Introduction

Comparing the surface properties of highly dispersed transition-metal particles with dimensions ranging from 1 nm to 10 nm in the liquid phase to the surface properties of their supported catalysts is an interesting investigation. Usually, metal catalysts are applied in the liquid phase; thus an understanding of their surface nature is important in the context of their liquid phase catalytic applications. It is therefore desirable to investigate the metal catalysts in the liquid phase, the same environment in which their catalytic properties are evaluated.^1–7^

So far, many research groups^1,4–7^ have investigated the surface chemical compositions, structures and properties of colloidal metals and their alloy dispersions by carrying out simple spectroscopic investigations of gaseous adsorbates, predominantly CO, on colloidal metal surfaces. Their noble works are enhancing our knowledge of colloidal metal surface chemistry and providing versatile applications of these metal particles to catalysis.^8^

Due to the high infrared extinction coefficient of adsorbed CO, it is the most widely investigated adsorbate in catalytic surface science and chemistry for the structural analysis of metal surfaces and their alloys. Using spectroscopy to structurally characterize single crystal surfaces with adsorbed CO is an established method that relies on the surface properties of small metal particles. These properties have in particular allowed the use of infrared spectroscopy to probe the surface compositions and carry out structural studies of highly dispersed supported metal catalysts.

A relatively high infrared extinction coefficient can be obtained in transmission mode due to the lower scattering of infrared radiation even at high particle concentration, which has made this technique particularly amenable to use. Another advantage of the study of CO adsorbed on colloidal metals is that adsorption equilibrium is reached rapidly during the addition of the gaseous adsorbate to the liquid dispersion, due to the absence of diffusion barriers that may exist in solid samples.^9^

The application of infrared spectroscopy to study colloidal metal surfaces has been developed by many research groups, and the spectra of CO adsorbed on hydrosols of platinum, palladium^10–12^ and rhodium,^8^ and on organosols of nickel,^13^ palladium,^1,3,8,14^ platinum,^2,4,8,15–17^ ruthenium^8^ and palladium–copper alloys^18–20^ have been reported.

Studies of surfaces covered by the stabilizer poly(vinylpyrrolidone) (PVP), their bonding manner and number of stabilizer molecules attached per particle are still in progress. It is well accepted that PVP molecules are weakly coordinated to metal particles *via* oxygen atoms through multiple sites where partial electron transfer occurs from PVP to the attached metal atom.^21–23^ This coordination bond is very weak and allows for an easy interchange with a suitable ligand.^24^ Some researchers have also suggested that only one PVP molecule is attached per nanoparticle.^25^ Additionally, PVP-protected particles can easily catalyze a variety of reactions without substrate hindrance.^21,26–29^ From these reports, we assume that a sufficient amount of surface is exposed on PVP-protected Pt (Pt:PVP) nanoparticles (NPs). Moreover, the small CO molecule could easily avoid any hindrance by the bulky PVP molecule and hence come into contact with the Pt surface, and could create more exposed space by replacing some parts of the PVP coordinated to the metal. These investigations suggest that CO can adsorb onto colloidal metal surfaces in a manner similar to that onto the corresponding clean system.

Although it is a challenge to prepare and stabilize very small particles, a study of size-dependent properties, especially surface properties, requires monodispersed particles in a range of sizes to be available, preferably stabilized by the same polymer.^30,31^ In the current work, we acquired IR spectra of ^12^CO adsorbed onto an organosol of PVP-stabilized platinum NPs with approximate mean particle diameters of 1.4 nm, 1.8 nm, 3.2 nm, 3.9 nm and 4.8 nm and also investigated the effect of the size of the particle on its surface properties. Metal particles in this size range are often not discerned using electron microscopy whether or not the particles are well-formed with faceted surfaces. Thus a surface sensitive technique must be applied in order to describe the nature of the particle surface. An IR flow cell was designed with the help of a syringe and Teflon tube in order to allow continuous collection of IR spectra of adsorbed CO at a range of coverages on the colloidal metal surfaces.

## 2. Experimental

### 2.1 Chemicals and materials

Hexachloroplatinic acid hexahydrate (H_2_PtCl_6_·6H_2_O, Sigma Aldrich), PVP (average molecular weight: 40 000; Sigma Aldrich), sodium borohydride (NaBH_4_, Sigma Aldrich), ethylene glycol (Tokyo Chemical Industry), ethanol (Merck), and analytical grade CO gas were used. Deionized water was used to prepare aqueous solutions.

### 2.2 Preparation of PVP-stabilized Pt NPs

#### 2.2.1 Microfluidic reduction with BH_4_^−^ (sample **a**, *d*_TEM_ = 1.4 nm)

Pt:PVP particles with diameters of 1.4 nm were produced according to our previous protocol.^32,33^ In brief, an ice-cooled Pt precursor (4 mM, 35 mL) and NaBH_4_ solution (20 mM, 35 mL) both containing 80 mM PVP (with respect to monomer) were passed through a micromixer (interdigital triangular) at room temperature with the help of syringe pumps. After discarding the initial few milliliters, the eluent was collected to a conical flask with continuous stirring for an hour. Finally, the produced Pt:PVP was purified by passing it through a hemodialyzer according to our previous report^34^ to remove the inorganic ions and other impurities. The purified Pt:PVP was freeze dried, and the resulting sample was pulverized and stored in an air-tight desiccator.

#### 2.2.2 Ethylene glycol reduction with NaOH (sample **b**, *d*_TEM_ = 1.8 nm)

Pt:PVP particles with diameters of 1.8 nm were produced according to a reported protocol^32^ but with a little modification. A mass of 155 mg (0.3 of a millimole) of vacuum-dried (40 °C temperature and 10 mm Hg pressure) H_2_PtCl_6_·6H_2_O and 180 mg of NaOH (4.5 millimoles) were placed into a 50 mL round-bottom flask. A volume of 15 mL of ethylene glycol was also added to this flask, and the resulting solution was stirred for 3 h at room temperature to ensure complete dissolution of the NaOH. Then the solution was heated at 140 °C for ∼2 h with vigorous stirring using a magnetic stirrer under flowing nitrogen gas. A mass of 1332 mg (12 millimoles with respect to monomer) of PVP (PVP-monomer:Pt = 40 : 1) was added to this hot solution and left stirring overnight. Finally, the produced Pt:PVP was purified to remove ethylene glycol and other inorganic impurities, as was done for sample **a**, and was then stored.

#### 2.2.3 Ethanol reduction (sample **c**, *d*_TEM_ = 3.2 nm and sample **d**, *d*_TEM_ = 3.9 nm)

Sample **c** and sample **d** were prepared according to our published protocols.^35^ A mother solution was prepared by adding 52 mg (0.1 of a millimole) of H_2_PtCl_6_·6H_2_O and 444 mg (0.4 of a millimole with respect to monomer) of PVP into a flask containing 90 mL of 90% EtOH. To prepare Pt:PVP particles with diameters of 3.2 nm, 60 mL of this mother solution was taken into a 100 mL round-bottom flask and refluxed at 90 °C for 3 h under air. Within 30 min, the yellowish solution turned black, and the reaction was continued for 3 h at which point it reached completion.

To prepare a sample of Pt:PVP particles with diameters of 3.9 nm, the Pt:PVP particles with diameters of 3.2 nm were used as seeds. A volume of 30 mL of as-produced sample **c** solution and the remaining 30 mL of the mother solution were taken into a 90 mL round-bottom flask. The resultant solution was refluxed at 90 °C for 3 h under air to produce sample **d**. Finally, to remove the ethanol and other impurities, the solutions were dialyzed as was done for sample **a** and then stored.

#### 2.2.4 Ethylene glycol reduction without NaOH (sample e, *d*_TEM_ = 4.8 nm)

A mixture of 31 mg of H_2_PtCl_6_·6H_2_O and 120 mg of PVP (PVP-monomer/Pt molar ratio of 18) was prepared in 4 mL of ethylene glycol and vacuum dried (40 °C temperature and 10 mm Hg pressure). This solution was rapidly added to 10 mL of boiling ethylene glycol at 200 °C under vigorous stirring, and boiling and stirring were continued for another 5 min. The resulting solution was cooled to room temperature, and then a mass of 148 mg of PVP was added to this cooled solution, which was then stirred overnight. Finally, the produced Pt:PVP was purified to remove ethylene glycol and other inorganic impurities as was done for sample **a** and then stored.

## 3. Characterization of the produced Pt:PVP NPs

### 3.1 UV-visible spectroscopy

UV-visible optical spectra of dispersed Pt:PVP in deionized water were recorded using a spectrophotometer (UV-1800, Shimadzu) at room temperature.

### 3.2 Transmission electron microscopy

The core sizes of the Pt:PVP were measured by using a transmission electron microscope (TEM, H-9500, Hitachi) operated at 220 kV with magnifications from 50 000 to 200 000. For TEM measurements, carbon-coated copper grids were used for sample casting. An approximately 0.25 mM concentrated Pt:PVP dispersion in EtOH was prepared and drops of this dispersion were drop-casted onto grids placed on filter paper (one drop per grid). After complete drying at room temperature, the grids were placed in TEM for the measurements.

### 3.3 X-ray diffractometry (XRD)

A D8 ADVANCE (Bruker) diffractometer at 40 kV and 40 mA and with Cu Kα radiation (1.5418 Å) was used to acquire the X-ray diffraction (XRD) patterns of the prepared and purified Pt NPs. The finely powdered freeze-dried sample (∼20 mg) was used for this measurement. The obtained diffraction patterns were analyzed using the TOPAS-4 program.

### 3.4 Fourier transform IR (FTIR) spectroscopy

**Scheme 1:**
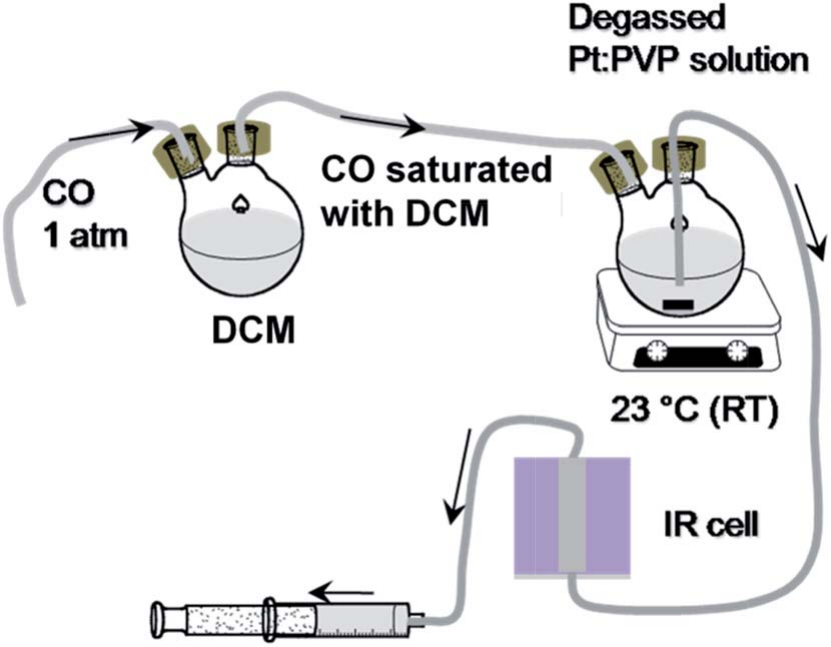
Acquisition of FTIR spectra of CO adsorbed on the Pt NP surface.

The FTIR spectroscopic measurements were taken according to the following steps. (a) Instrumentation: an FTIR spectrometer (Spectrum 10, PerkinElmer) operating at a resolution of 4 cm^−1^ was used to record spectra of the ^12^CO molecules adsorbed onto Pt:PVP organosol. (b) Sample preparation: to allow the areal intensities of spectra to be compared, Pt:PVP particles (0.6 mmol with respect to Pt metal) were dispersed in 5 mL of dichloromethane (CH_2_Cl_2_, DCM). The prepared organosols were transferred into a septum-sealed, two-neck round-bottom flask and deaerated by performing freeze–pump–thaw cycles, specifically to remove dissolved O_2_, CO_2_, water vapor, *etc.* Then, one end of the two-neck flask was connected to DCM saturated with CO and another end was connected to an IR cell through a Teflon tube. The prepared solution was then purged with DCM saturated with CO at atmospheric pressure and at a temperature of 23.0 ± 0.5 °C for about 10 minutes to attain the adsorption equilibrium. (c) Measurements: in purging condition, organosols were transferred *via* a Teflon tube to the IR cell by pulling out a syringe connected to the other end of the IR cell as shown in Scheme 1. Finally, the FTIR spectra were recorded in transmission mode and later converted to absorbance mode. For better resolution, 40 scans were averaged and background spectra were removed.

## 4. Results and discussion

### 4.1 Optical spectra for NP formation

**Fig. 1:**
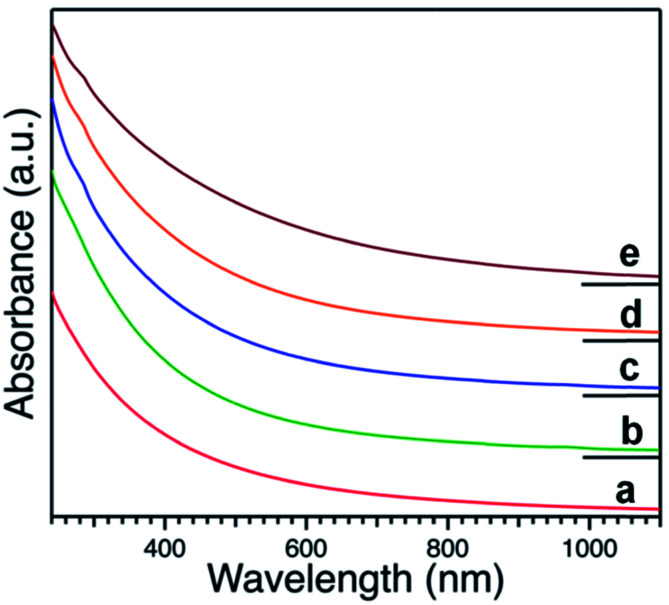
UV-visible spectra of hydrosols of PVP-stabilized Pt NPs with different diameters as indicated in Table 1.

Representative UV-Vis spectra of produced Pt:PVP hydrosols normalized at a concentration of 0.2 mM with respect to Pt metal measured by ICP are shown in Fig. 1. All of the optical spectra exhibited profiles with a tendency to exponentially increase in the short wavelength with no obvious absorption peak due to intra- and inter-band optical transitions, as have been reported for Pt NPs.^32,33,36–38^

### 4.2 Size distribution of Pt NPs

**Fig. 2:**
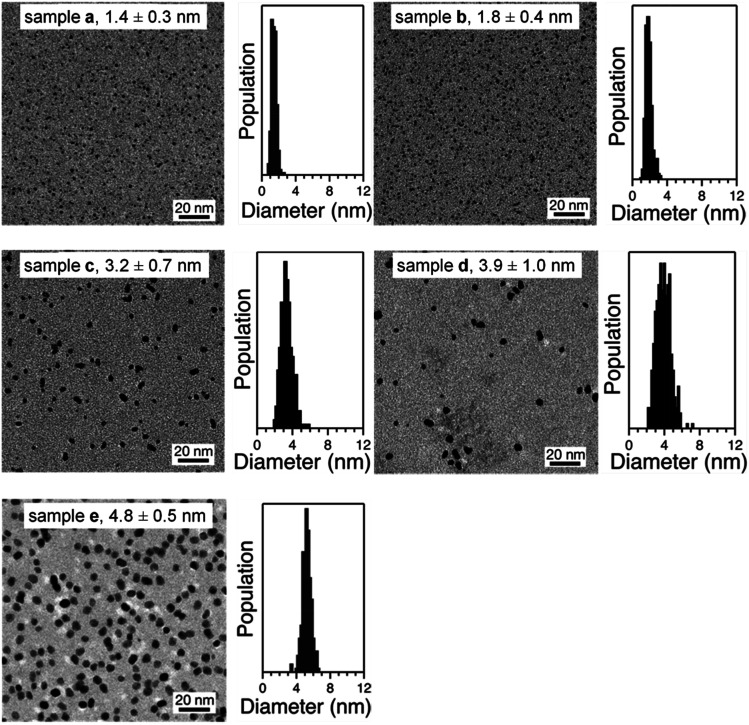
Representative TEM images and core size distributions of PVP-stabilized Pt NPs.

Representative TEM micrographs of purified Pt:PVP are shown in Fig. 2. All of these samples were found to be comprised of spherical particles, even after taking their HRTEM images. From the TEM micrographs, the diameters of more than 350 Pt NPs were measured in each sample; and the average diameters and their size distributions were calculated using a Gaussian function and are shown in Table 1.

**Table 1:** Average diameter (*d*_TEM_) values of samples of produced Pt:PVP particles with their size distributions

Sample	Average *d*_TEM_ (nm)
a	1.4 ± 0.3
b	1.8 ± 0.4
c	3.2 ± 0.7
d	3.9 ± 1.0
e	4.8 ± 0.5

**Fig. 3:**
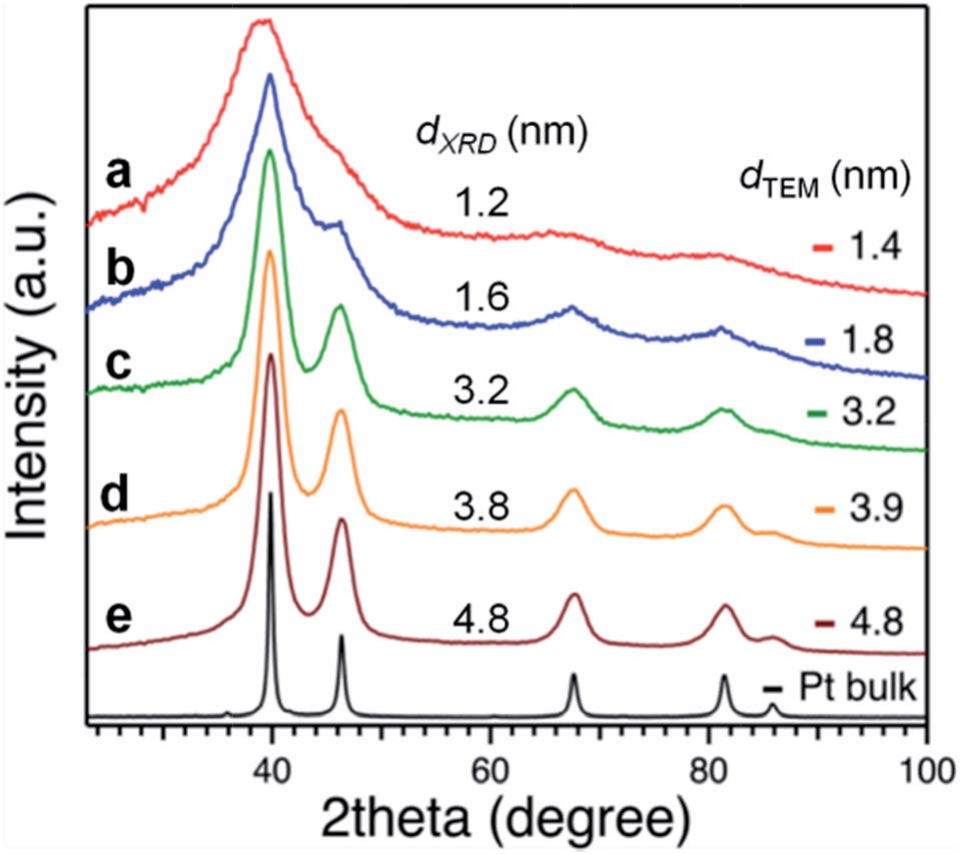
XRD patterns of Pt:PVP samples and bulk Pt.

Applying the Scherrer formula to the Pt(111) diffraction peaks, the average diameters of the Pt crystallites (*d*_XRD_) were evaluated as shown in Fig. 3. All of the Pt NPs had an fcc crystal geometry. The crystallite sizes determined from XRD analysis were comparable to the average diameters determined from the TEM analysis (Fig. 2).

### 4.3 Surface structure of Pt NPs

**Fig. 4:**
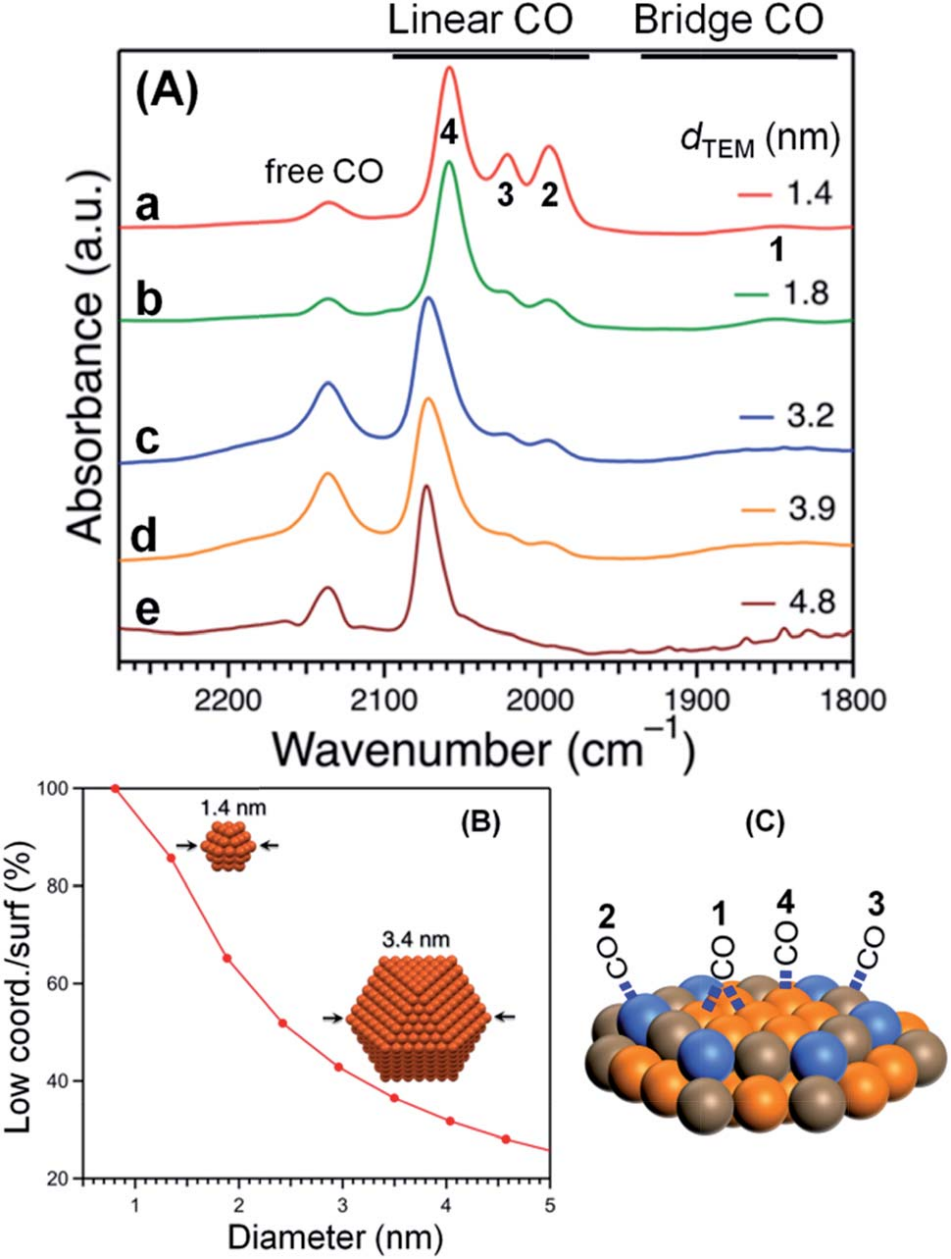
(A) FTIR spectra of CO adsorbed onto Pt:PVP. (B) Low-coordination sites/surfaces as a function of Pt NP diameter considering a cuboctahedral model. (C) The assignment of peaks.

The acquired normalized FTIR spectra of the CO molecule on Pt:PVP samples (Pt-atom/PVP-monomer = 1/40) are shown in Fig. 4(A). The organosols were purged for 20 min before taking the spectral measurements. Each of the five spectra showed five peaks with different intensities, which are shown in Table 2 with their plausible assignments.

**Table 2:** The probable assignments of the five peaks and their bonding modes

Peak No.	Wave number (cm^−1^)	Bonding mode	Assignment
1	1850	Bridge between two atoms	Due to a bridge site between two Pt atoms on the terrace,^10^ very weak peak
2	1994	On top/single atom	CO adsorbed on a single Pt atom near/along the edges^40,41^ due to stronger adsorption energy for smaller particles
3	2021	On top/single atom	CO adsorbed on a single Pt atom near the vertices^40,41^ due to stronger adsorption energy for smaller particles
4	2058	On top/single atom	CO adsorbed on a single Pt atom on the terrace,^10,40,41^ strongest among the peaks due to the higher population with increasing particle size
5	2137	Free CO	Free CO dissolved in CH_2_Cl_2_ (ref. 3 and 8)

Considering a cuboctahedral model, smaller particles have fewer terrace atoms than do larger particles and thus have more edge and vertex atoms than do larger particles. The relative intensities of the peaks at 1994 cm^−1^ (peak 2) and 2021 cm^−1^ (peak 3) were very similar and were assigned to the edges and vertices, respectively, as shown in Fig. 4(B) and (C).

The coordination number of each adsorption site controls the extent of interaction with CO molecules. Top and bridge binding sites near the edges and vertices of the NP facets were indicated to be the most favorable adsorption sites for CO molecules, as was observed in our previous investigations ,^40,41^ due to the adsorption energies being stronger near the edges and vertices of NPs. However, support effects weaken the adsorption energies, which are lower for larger NPs, but are significant for smaller NPs.^40^

**Fig. 5:**
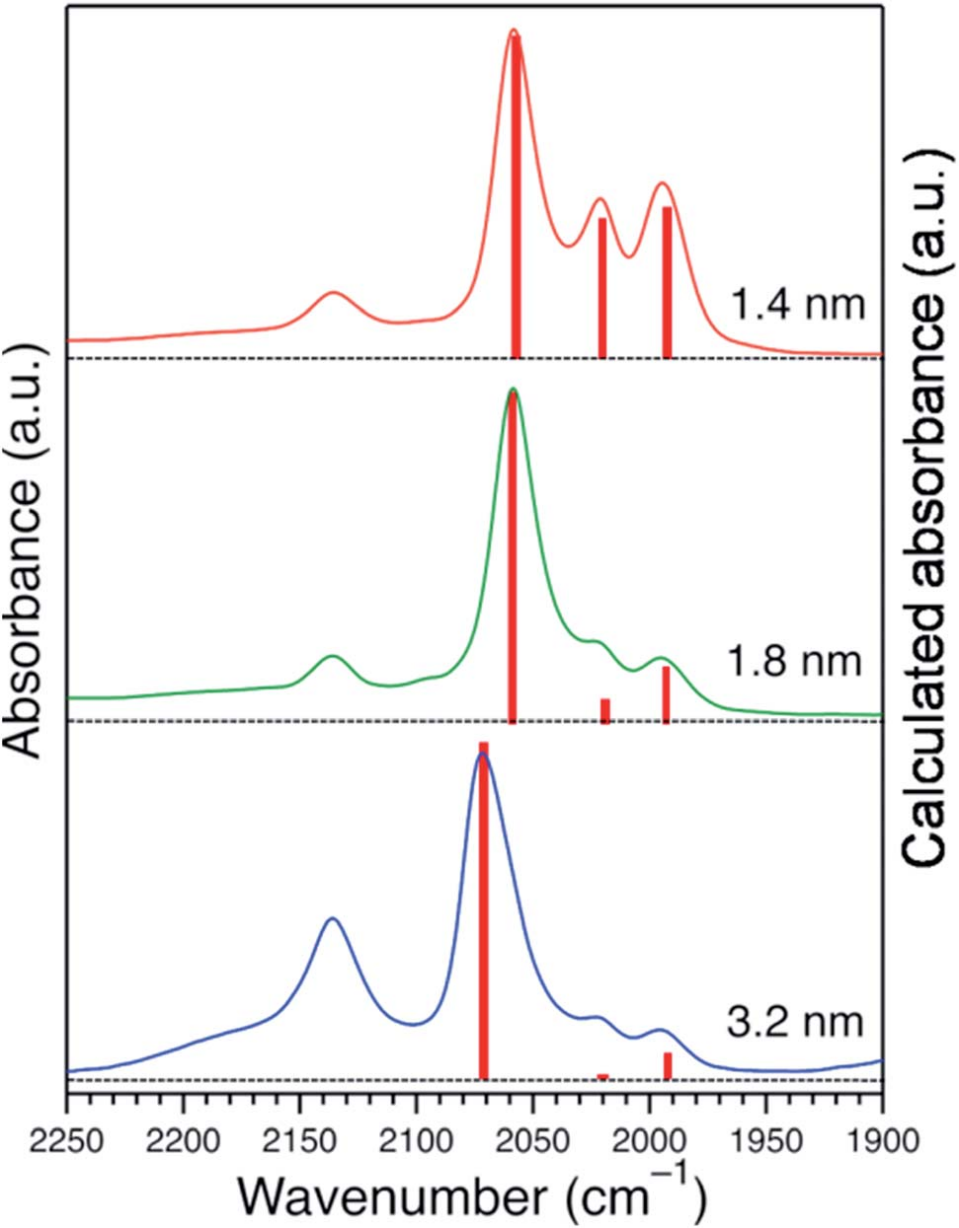
A comparison of the FTIR spectra of adsorbed CO obtained experimentally and *via* calculations. The continuous lines represent the experimental results and the bars represent the calculated results.

Assuming a cuboctahedron structure for all Pt NPs, to calculate the population of surface sites of Pt NPs, the relative IR intensities of the Pt:PVP samples were used, as was the equation*I*_x_ = *N*_x_ × *C*_x_ × *P*_x_for calculating the IR intensity at site x (*I*_x_). In this equation, *N*_x_ = number of atoms of site x, *C*_x_ = coverage of CO, *P*_x_ = photo-absorption probability, which was the same for all surface sites. The saturation coverage of CO for Pt:PVP particles with diameters of 1.4 nm was used to explain the CO coverage for other Pt:PVP samples. From the 100% coverage for the Pt NPs with diameters of 1.4 nm, the ratio of the saturation coverage for the terrace to that of the corner to that of the edge, *i.e.*, *C*_T_ : *C*_C_ : *C*_E_, was determined to be 1.0 : 0.3 : 0.1. Then this ratio was used to calculate the relative intensities of the Pt:PVP samples, which were in good agreement with the experimental results as shown in Fig. 5.

The terrace CO peak positions (peak 4) were red shifted^1,8^ and the extent of red shifting increased with decreasing particle size. This increased red shift was ascribed to the increased electron density on the Pt metal due the partial electron donation from PVP to the Pt metal.^32,39^ As the particles size was decreased, there were more low-coordination sites; this result indicated the occurrence of more efficient electron transfer to ^12^CO from the increased electron density of smaller Pt particles *via* electron back-donation from the Pt surface to the 2π* orbital of CO, with this back-donation weakening the C–O bond of the CO molecule.^42^ This efficient electron transfer from smaller Pt:PVP particles to ^12^CO molecules was ascribed to the ability of the high-lying orbitals to accommodate excess electric charge due to the LUMO levels of the free Pt particles becoming higher in energy with a decrease in size.^27,43^

**Fig. 6:**
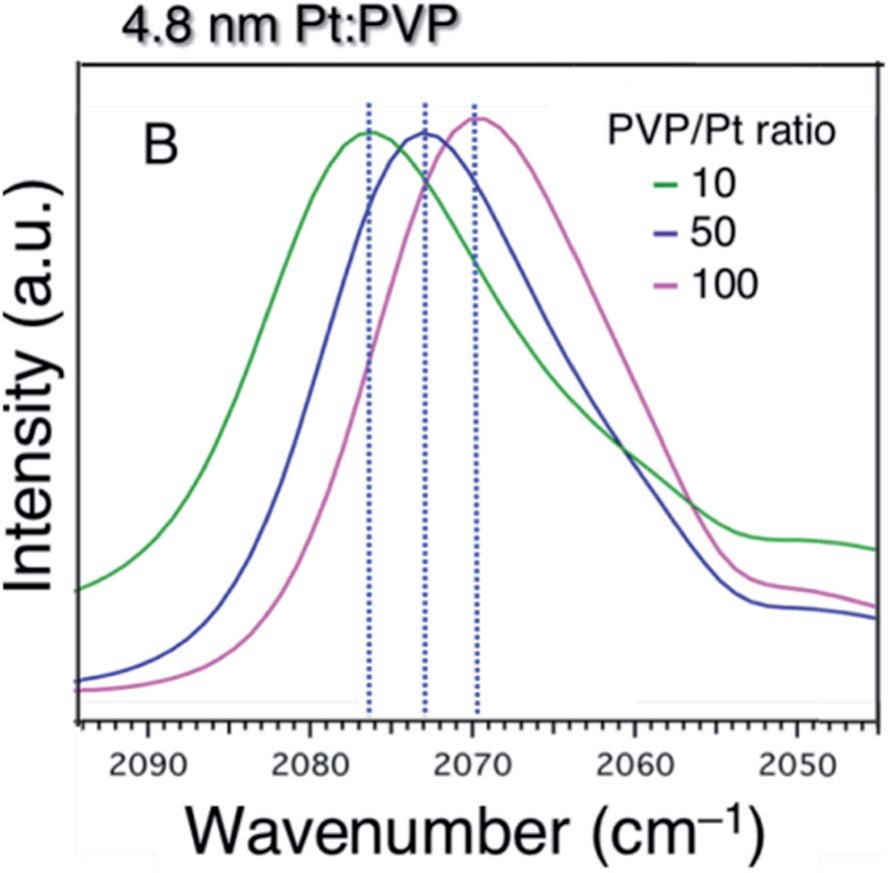
FTIR spectra of 4.8 nm-diameter Pt:PVP particles subjected to 20 min of purging with CO and consisting of various molar ratios of PVP monomer to Pt atoms as indicated.

For further confirmation of the donation of electron from PVP to Pt particles, the charge state of the Pt core was studied as a function of PVP concentration. Fig. 6 shows the acquired FTIR spectra of ^12^CO adsorbed on the 4.8 nm-diameter Pt:PVP particles dispersed in CH_2_Cl_2_ containing different amounts of PVP. The vibrational frequency of the adsorbed ^12^CO monotonically red-shifted from 2083 cm^−1^ to 2070 cm^−1^ as the [PVP-monomer]/[Pt-atom] ratio was increased from 10 to 100. This result clearly indicated that additional PVP contributed more electronic charge to the Pt cores of the 4.8 nm-diameter Pt:PVP particles dispersed in CH_2_Cl_2_, and hence provided an important indication of the direct role played by PVP in regulating the electronic structures of the Pt particles in addition to its role as a stabilizer.

**Fig. 7:**
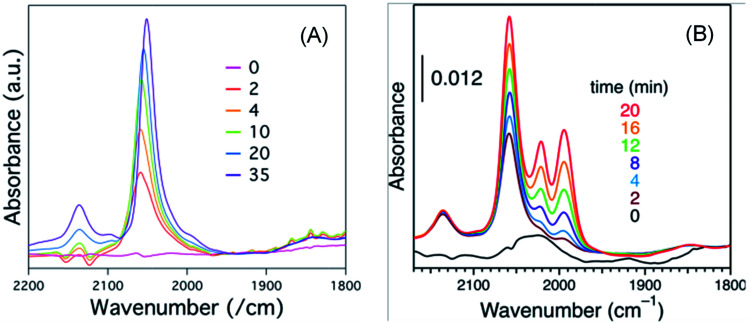
Sets of time-course FTIR spectra of CO adsorbed on 1.4 nm-diameter Pt:PVP particles acquired after (A) 6.0 h or (B) 0.5 h of dissolution in DCM.

Solvent played an important role on the geometric and electronic structure of the NPs. Some solvents like DCM, a very strong solvent, might restructure the NP surface in a way that could not be observed from XRD, TEM or UV-visible spectroscopy analyses. Many other research groups have also reported a similar procedure as we did for acquiring FTIR spectra of CO molecules adsorbed on Pt NPs,^14^ but our results differed somewhat. Perhaps, the NP preparation condition or degassing process or the total time from dissolution to CO adsorption and measurement was very important. After 6 hours of dissolution time, peaks from about 1994 cm^−1^ to 2021 cm^−1^ (peak 2 and peak 3) were absent; and with increasing coverage of CO, the terrace peak position gradually red shifted, as shown in Fig. 7(B). However, in case of freshly prepared organosol, there was no such shift of the terrace peak, and also the edge and corner peaks were present, as shown in Fig. 7(A). Perhaps some structural rearrangement occurred during the longer dissolution of Pt:PVP due to the strong effect of DCM and hence we did not get results similar to those of the fresh Pt:PVP solution in DCM.

## 5. Conclusions

We investigated the surface structures of colloidal Pt:PVP particles with diameters between 1.4 nm and 4.8 nm by acquiring FTIR spectra of adsorbed ^12^CO molecules. Two new bands at 2021 cm^−1^ and 1994 cm^−1^ were observed relating to the proportion of corner and edge atoms on the surface when considering a cuboctahedral model. Also, the terrace CO peak position was observed to be relatively red shifted, which was attributed to electron donation from PVP to Pt NPs and which indicated that the electronic structure of the NPs can be regulated by the PVP stabilizer. In addition, DCM solvent was found to affect the geometric structure of the Pt NPs when the NPs were subjected to prolonged dissolution in the solvent. Due to the high sensitivity of FTIR spectroscopy for adsorbed CO molecules on the NP surfaces, by taking careful measurements we were able to calculate the surface site populations of Pt NPs as well as their charge states.

## Conflicts of interest

There is no conflict of interest with others.

## Supplementary Material
